# Unusual Presentation of Cryptococcal Meningitis in an Immunocompetent Patient on Hemodialysis

**DOI:** 10.7759/cureus.91673

**Published:** 2025-09-05

**Authors:** Varshitha Tumkur Panduranga, Jacob Sabu, Bernard Brown, Khaleda Akter, Nikhita Kalra, Tanveen Dhallu, Helen Valsamis, Hamza Hamza, Fnu Veerban, Ibrahim Mohamed, Isha Puri, Muhammad Azhar, Mary Mallappallil

**Affiliations:** 1 Internal Medicine, State University of New York Downstate Health Sciences University, Brooklyn, USA; 2 Neurology, State University of New York Downstate Health Sciences University, Brooklyn, USA; 3 Internal Medicine, Kings County, Brooklyn, USA; 4 Internal Medicine/Neurology, State University of New York Downstate Medical Center, Brooklyn, USA; 5 Neurology, Kings County, Brooklyn, USA; 6 Nephrology, State University of New York Downstate Medical Center, Brooklyn, USA; 7 Medicine, State University of New York Downstate Health Sciences University, Brooklyn, USA; 8 Internal Medicine/Nephrology, State University of New York Downstate Health Sciences University, Brooklyn, USA; 9 Nephrology, New York City Health and Hospitals Corporation (NYCHHC) Kings County Hospital Center, Brooklyn, USA; 10 Medicine, State University of New York Downstate Medical Center, Brooklyn, USA

**Keywords:** burnt out diabetes, cryptococcal meningitis, cryptococcus neoformans (c. neoformans), immunocompetent, patients with hemodialysis

## Abstract

Cryptococcal meningitis (CM) is an invasive fungal disease that poses a global health threat, particularly in immunocompromised individuals. CM mainly occurs in HIV-positive patients and other immunocompromised patients and is rare in immunocompetent persons. The clinical presentation and disease course of CM in an immunocompetent patient remain poorly understood due to limited data. We present the case of a 60-year-old woman with multiple comorbidities, including hypertension, burnt-out diabetes mellitus, chronic kidney disease on hemodialysis, heart failure with preserved ejection fraction, who was brought to the emergency department with a change in her mentation. Initial evaluation suggested a differential of uremic encephalopathy, cerebrovascular accident, or hypertensive emergency. Despite hemodialysis and supportive care, the patient’s lethargy and confusion did not improve. Neuroimaging revealed leptomeningeal enhancement, and lumbar puncture confirmed the diagnosis of cryptococcal meningitis through positive cerebrospinal fluid (CSF) PCR and cryptococcal antigen testing. Notably, the patient had no obvious immunosuppressive conditions, highlighting an atypical presentation of CM in an apparently immunocompetent host. This case underscores the importance of considering CM in the differential diagnosis of altered mental status, even in patients without overt immunosuppression. Understanding such atypical presentations is crucial for timely diagnosis, guiding effective treatment strategies, and improving outcomes in immunocompetent individuals affected by CM.

## Introduction

Cryptococcal meningitis (CM) is a severe and fatal fungal infection that continues to be a global health concern, causing significant illness and death, especially among immunocompromised individuals [1]. It is caused by encapsulated yeasts called Cryptococcus neoformans. CM can affect various organ systems; its neurological involvement is the most devastating. The disease predominantly affects immunocompromised populations, including individuals with HIV, organ transplant recipients, and patients receiving corticosteroids or other immunosuppressive therapies. However, cases have been documented in immunocompetent individuals, though these remain relatively rare and underreported [2]. Cryptococcal meningitis (CM) can be challenging to diagnose due to its often subacute and nonspecific presentation. It should be considered in any patient presenting with headache, altered mental status, or focal neurological deficits, particularly those who are immunocompromised. Prompt recognition is critical, as early diagnostic testing and initiation of empiric antifungal therapy are essential for improving outcomes. Diagnosis is generally made with cerebrospinal fluid (CSF) analysis, using microscopy, culture, and cryptococcal antigen testing, offering high sensitivity. However, despite advances in antifungal therapy, CM continues to carry a high mortality rate, highlighting the need for vigilance and timely intervention [3].

## Case presentation

A 60-year-old woman with a past medical history of hypertension, hyperlipidemia, type 2 diabetes mellitus (DM) with DM retinopathy but now off medications, congestive heart failure with preserved ejection fraction (HFpEF), and end-stage renal disease (ESRD) on hemodialysis for approximately one and a half years was brought to the emergency department (ED) for evaluation of altered mental status (AMS). At baseline, she was alert, awake, and oriented to time, place, and person. She resided in a nursing home, where she was found to be lethargic, non-verbal, and had decreased oral intake for the past three days. It was also noted that she had missed a single dialysis session the previous day since she was sent to the hospital. In the emergency department, the patient was afebrile, with a blood pressure of 201/106 mmHg, heart rate of 77 bpm, respiratory rate of 20 breaths per minute, and oxygen saturation of 90% on room air. Physical exam was unremarkable with no signs of nuchal rigidity, Kernig sign or focal neurological deficits. The patient was not noted to be on any long-term corticosteroids or immunosuppressive medications. Laboratory tests (Table 1) were remarkable for a creatinine of 5.15 mg/dL, blood urea nitrogen (BUN) of 58 mg/dL, an anion gap of 22, proBNP of 70,000 pg/mL, lactate of 1.5 mmol/L, and an elevated troponin of 186 ng/L, which later down-trended to 171 ng/L. Urinalysis revealed calcium crystals. Imaging studies included a chest X-ray showing a right-sided pleural effusion, and a CT head without intravenous contrast showed no acute intracranial pathology. A CT scan of the abdomen and pelvis revealed no acute intra-abdominal abnormalities. She was initially treated with intravenous furosemide and admitted with a working diagnosis of uremic encephalopathy versus hypertensive emergency.

**Table 1:** Laboratory Tests ALT: Alanine transaminase; AST: Aspartate transaminase

Test	Result	Reference Range
Sodium (mmol/L)	138	135 – 145
Potassium (mmol/L)	4.1	3.5 – 5.0
Chloride (mmol/L)	97	96 – 106
BUN (mg/dL)	58	7 – 20
Creatinine (mg/dL)	5.1	0.6 – 1.3
Glucose (mg/dL)	143	70 - 200
ALT (U/L)	6	7 – 56
AST (U/L)	11	10 – 40
Total Bilirubin (mg/dL)	0.8	0.1 – 1.2
Calcium (mg/dL)	8.1	8.5 – 10.5
Total Protein (g/dL)	6.7	6.0 – 8.3
Albumin (g/dL)	4.4	3.5 – 5.0
Magnesium (mg/dL)	2.59	1.7 – 2.2
Phosphorus (mg/dL)	6.6	2.5 – 4.5
eGFR (mL/min/1.73 m²)	8.6	>60
Troponin (ng/L)	186	<14
Pro-BNP (pg/mL)	70,000	<125
Lactic Acid (mmol/L)	1.2	0.5 – 2.2
TSH (µIU/mL)	2.8	0.4 – 4.0
WBC (×10³/µL)	9.94	4.5 – 11.0
RBC (×10⁶/µL)	4.84	4.2 – 5.9
Hemoglobin (g/dL)	13.3	12 -15
Hematocrit (%)	43.2	36 - 44
Platelets (×10³/µL)	270	150 – 450
Monocyte Count (×10³/µL)	0.37	0.2 – 0.8
Neutrophil Count (×10³/µL)	8.96	1.8 – 7.8
Lymphocyte Count (×10³/µL)	0.55	1.0 – 3.0
Eosinophil Count (×10³/µL)	0.02	0.0 – 0.5
Basophil Count (×10³/µL)	0.01	0.0 – 0.2
Immature Granulocyte Count (%)	0.3	0.0 – 0.4

The patient underwent hemodialysis on the day of admission and again two days later. Regarding her diabetes, the patient had a history of insulin-dependent type 2 diabetes mellitus; however, in recent months, particularly during her stay in the nursing home, she no longer required treatment. Her hemoglobin A1c remained within normal range without pharmacologic therapy, consistent with a phenomenon known as "burnt-out diabetes," commonly observed in patients on chronic dialysis. Hepatitis serologies were notable for positive hepatitis C, which was chronic, and resolved hepatitis B infection, the latter indicated by serologic evidence of natural immunity. Additional infectious workup was negative, including tests for HIV, syphilis, COVID-19, RSV, and influenza. Blood cultures showed no growth, and vitamin B12 and folate levels were within normal limits. During her hospital course, she developed increased work of breathing with use of accessory muscles and retractions, though her oxygen saturation remained in the high 90s. Arterial blood gas analysis revealed metabolic acidosis likely due to a combination of uremia and starvation ketoacidosis, with a compensatory respiratory alkalosis.

**Figure 1:**
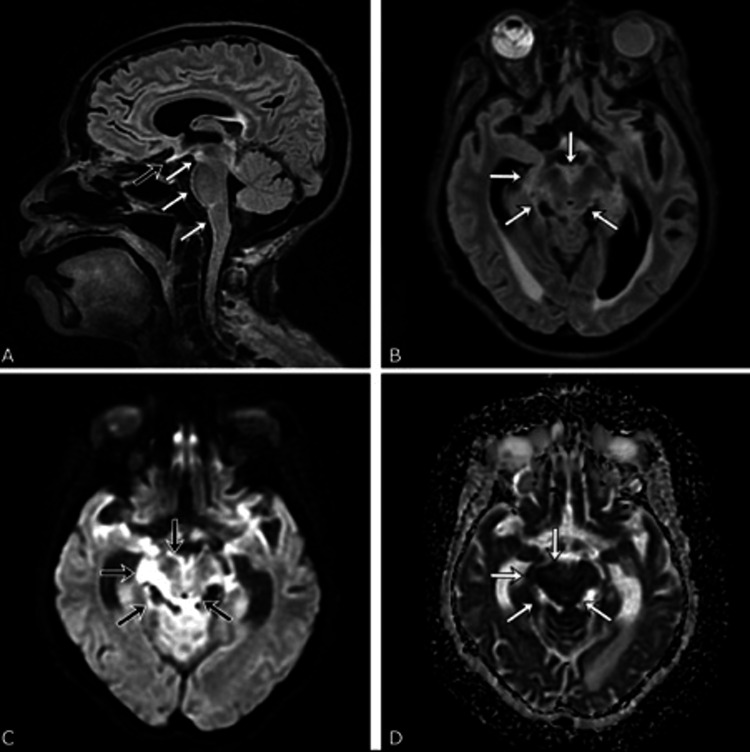
MRI without gadolinium. (A) FLAIR sequence hyperintensities are present on the optic nerve (black arrow) and other cranial nerves not shown here; a sugar-coating pattern of hyperintensity is seen on the midbrain, pons, and medulla (white arrows). (B) FLAIR abnormalities are coating the midbrain and perimesencephalic cisterns with greater prominence in the patient’s right. (C) DWI sequence showing restriction in the corresponding FLAIR lesions. (D) ADC sequence showing restriction correlation with DWI DWI: Diffusion-Weighted Imaging; ADC: Apparent Diffusion Coefficient

Further evaluation of her persistent AMS included an MRI brain (Figure 1), which showed hyperintensities present on multiple bilateral cranial nerves, midbrain, pons and medulla with signal abnormalities present in the midbrain and perimesencephalic cisterns concerning for leptomeningeal disease. A lumbar puncture (Table 2) revealed a normal opening pressure of 17.5 cmH₂O but showed elevated neutrophils, low glucose, and high protein. CSF PCR and cryptococcal antigen testing confirmed the presence of Cryptococcus neoformans, with a titer of 1:320. Empiric antibiotics with ampicillin, ceftriaxone, and vancomycin were initially started but subsequently transitioned to antifungal therapy with amphotericin B and fluconazole. Despite treatment, the patient’s condition continued to decline with worsening oral intake and respiratory distress, and she ultimately passed away.

**Table 2:** Lumbar Puncture (CSF) Results CSF: Cerebrospinal fluid

Test	Result	Reference Range
Opening Pressure (cmH₂O)	17.5	6 – 20
Fluid Appearance	Cloudy	Clear
Polymorphonuclear Cells (%, CSF)	76	≤8%
Lymphocytes (%, CSF)	7	40 – 90%
Monocytes (%, CSF)	17	15 – 60%
Total Cell Count (cells/µL)	100	0 – 5
Cryptococcus Titre	1:320	Negative
Glucose (mg/dL, CSF)	24	45 – 80
Protein (mg/dL, CSF)	196	15 – 45
PCR Panel, CSF	Cryptococcus neoformans	
Cryptococcal Antigen, CSF	Positive	Negative
IgG (mg/dL, CSF)	23.2	0.0 – 6.0
Albumin (mg/dL, CSF)	106.8	10 – 30

## Discussion

Cryptococcus neoformans and Cryptococcus gattii species are major causes of meningoencephalitis, often resulting in high mortality and significant long-term complications, especially in individuals with impaired T cell-mediated immunity - most frequently among those living with HIV [4]. However, the risk of cryptococcal infection is also elevated in solid organ transplant recipients and others with defects in cell-mediated immunity. Importantly, cases of cryptococcosis have also been reported in individuals who are apparently immunocompetent, but such presentations have been largely uncommon but clinically significant [4]. Despite available treatment, mortality remains high among affected populations. In recent years, significant progress has been made in the development of rapid point-of-care diagnostic tools and early detection methods for cryptococcal antigen in the bloodstream. These advances have facilitated the implementation of screening and pre-emptive treatment strategies, particularly targeting individuals with advanced HIV infection, to help prevent progression to clinical disease [5]. However, in immunocompetent individuals, cryptococcal infection remains difficult to detect due to the absence of routine screening in this population and the often non-specific and variable nature of its clinical presentation, which can delay diagnosis and treatment.

Each year, cryptococcal meningitis affects an estimated 1 million people globally, with a mortality rate exceeding 60% within the first three months of diagnosis [6]. Cryptococcus neoformans is the most common fungal pathogen responsible for central nervous system meningoencephalitis worldwide. Infection typically occurs through the inhalation of fungal spores or desiccated yeast from environmental sources [7]. After an initial asymptomatic infection in the lungs, the organism can enter the bloodstream and spread to various target organs such as the lungs, skin, and bones, often leading to lymphocytic meningitis [8]. The fungus evades the host immune system using various defence mechanisms like activation of superoxide dismutase, catalytic enzymes, and antioxidants adapting to the oxidative attack induced by the host, allowing penetration of the blood-brain barrier. The lack of complement activation in the cerebrospinal fluid presents as a favourable growth medium for Cryptococcus species [8].

The clinical presentation of cryptococcal meningoencephalitis is often subacute or chronic, with symptoms such as fever, malaise, and headache gradually progressing over one to two weeks. Patients may also experience neck stiffness, photophobia, vomiting, confusion, altered mental status, visual disturbances including reduced visual acuity, and hearing loss [9]. A definitive diagnosis requires microbiological analysis of cerebrospinal fluid (CSF) obtained via lumbar puncture. Compared to bacterial or tuberculous meningitis, the inflammatory response in cryptococcal infection is typically less pronounced, especially in immunocompromised individuals. CSF findings often include a mildly elevated white blood cell count (usually <50 cells/µL) with a predominance of mononuclear cells, modest protein elevation, and low or normal glucose levels [10]. Notably, up to 25-30% of culture-confirmed cases may present with a normal CSF profile, making diagnosis particularly challenging [11]. The diagnosis of cryptococcal disease is confirmed through microbiological methods, including microscopy, culture, and antigen testing, which are significantly more sensitive than diagnostic tests for most other central nervous system infections [2]. Three proven anti-fungal therapies for cryptococcal infections that have reliable efficacy are intravenous amphotericin B, oral flucytosine and oral fluconazole [2].

Cryptococcal meningitis (CM) in immunocompetent individuals is relatively uncommon, though emerging evidence suggests it may be more prevalent than previously thought, with some studies reporting up to 20% of cases occurring in this population [1]. The underlying risk factors in otherwise healthy individuals remain poorly understood, making it essential to investigate why a disease typically seen in immunosuppressed patients also affects young, seemingly healthy adults. In the absence of HIV or organ transplantation, certain comorbidities, such as cirrhosis, chronic heavy alcohol use, poorly controlled type II diabetes, and autoimmune disorders, have been identified as potential risk factors [12]. Although the patient had a history of diabetes, she achieved adequate glycemic control without the need for medications following the initiation of hemodialysis, making diabetes an unlikely contributing risk factor in this case. Less known and reported is immune dysfunction in those with kidney failure with possible shifts from lymphoid to myeloid cell lineage underlying uremia-associated immunological change, an alteration which is not reversed by renal replacement therapy [13]. Uremia-based immune dysfunction as the cause for her infection was supported by her low lymphocyte count.

The clinical presentation of cryptococcal meningitis may differ between immunocompromised and immunocompetent individuals; research specifically examining its features and outcomes in the latter group remains limited [14].

## Conclusions

Cryptococcal meningitis is a rare diagnosis in immunocompetent individuals, and its often non-specific, subacute presentation can make timely recognition particularly challenging. This case underscores the need to maintain a broad differential diagnosis when evaluating altered mental status, including the consideration of cryptococcal infection even in patients without common causes of immunosuppression. Early identification and prompt initiation of appropriate antifungal therapy are crucial to reducing morbidity and mortality.
